# Weighted residual network for SAR automatic target recognition with data augmentation

**DOI:** 10.3389/fnbot.2023.1298653

**Published:** 2023-12-19

**Authors:** Junyu Li, Cheng Peng

**Affiliations:** School of Electrical and Mechanical Engineering, Hefei Technology College, Hefei, China

**Keywords:** weighted residual network, data augmentation, synthetic aperture radar (SAR), automatic target recognition (ATR), deep learning—artificial intelligence

## Abstract

**Introduction:**

Decades of research have been dedicated to overcoming the obstacles inherent in synthetic aperture radar (SAR) automatic target recognition (ATR). The rise of deep learning technologies has brought a wave of new possibilities, demonstrating significant progress in the field. However, challenges like the susceptibility of SAR images to noise, the requirement for large-scale training datasets, and the often protracted duration of model training still persist.

**Methods:**

This paper introduces a novel data augmentation strategy to address these issues. Our method involves the intentional addition and subsequent removal of speckle noise to artificially enlarge the scope of training data through noise perturbation. Furthermore, we propose a modified network architecture named weighted ResNet, which incorporates residual strain controls for enhanced performance. This network is designed to be computationally efficient and to minimize the amount of training data required.

**Results:**

Through rigorous experimental analysis, our research confirms that the proposed data augmentation method, when used in conjunction with the weighted ResNet model, significantly reduces the time needed for training. It also improves the SAR ATR capabilities.

**Discussion:**

Compared to existing models and methods tested, the combination of our data augmentation scheme and the weighted ResNet framework achieves higher computational efficiency and better recognition accuracy in SAR ATR applications. This suggests that our approach could be a valuable advancement in the field of SAR image analysis.

## 1 Introduction

Due to its ability to operate independently of atmospheric and sunlight conditions, synthetic aperture radar (SAR) offers advantages over optical remote sensing systems. Automatic target recognition (ATR) is a crucial application of SAR systems, traditional techniques relied on handcrafted features such as the shape, size, and intensity of objects in the images (Oliver and Quegan, 2004). However, these techniques faced limitations as they required manual feature extraction and were susceptible to variations in conditions, object orientations, and configurations Wu et al. (2023a) and Yuan et al. (2023). In recent years, numerous approaches have emerged with the advancement of learning algorithms such as generative neural networks, multilayer autoencoders (Wu et al., 2022), long short-term memory (LSTM), and highway unit networks (Deng et al., 2017; Lin et al., 2017; Song and Xu, 2017; Zhang et al., 2017). However, it is important to note that even state-of-the-art machine learning algorithms may encounter challenges when applied to SAR ATR, such as the limited availability of training samples and the issue of model overfitting.

To address these challenges, Chen et al. (2016) have introduced all-convolutional networks (A-ConvNets) as a solution, reducing the number of free parameters in deep convolutional networks and thus mitigating the overfitting problem caused by limited training images. Furthermore, several SAR image data augmentation methods have been proposed in recent years, such as the works by Zha (1999), Ding et al. (2016), Wagner (2016), Xu et al. (2017), and Pei et al. (2018a), aiming to tackle the issue of limited training data.

In order to enhance the training data for SAR target recognition, several methods have been proposed. Zha (1999) suggested generating artificial negative examples by permutating known real SAR images to increase the dataset size. Wagner (2016) utilized positive examples to improve robustness against imaging errors. Pei et al. (2018a) developed a multi-view deep learning framework that generates a large amount of multi-view SAR data for training. This approach expands the training dataset by incorporating the spatial relationships between target images, resulting in improved recognition accuracy. Additionally, techniques such as suppressing speckle noise through fusion filters (Xu et al., 2017) and adding simulated speckle noise with varying parameters to training samples (Ding et al., 2016) were employed to enhance the SAR image data.

Among deep learning networks, Convolutional Neural Networks (CNNs) appear to be the most popular choice for SAR target recognition (Chen et al., 2016). However, severe model overfitting related to deep CNNs in SAR ATR was observed, leading them to propose an alternative solution called all-convolutional networks (A-ConvNets) to reduce the number of free parameters. A-ConvNets consist of sparsely connected layers instead of fully connected layers, providing a means of adjusting the model training process by improving network architecture.

There have been additional studies combining CNNs with assistant approaches, particularly in the context of data augmentation (Zhang et al., 2022; Wu et al., 2023b). The data augmentation methods used in SAR ATR can be broadly categorized into spatial information-related methods (Wagner, 2016; Pei et al., 2018a) and speckle noise-related methods (Xu et al., 2017). For spatial information-related approaches, Pei et al. (2018a) proposed a multiview deep learning framework that generates a large amount of multiview SAR data. This includes combinations of neighboring images with different azimuth angles but the same depression angle. By expanding the training dataset through this multiview SAR generation system, the spatial relations among target images are taken into account, resulting in higher model accuracy. Another typical method involves generating artificial images through distortion and affine transformation (Wagner, 2016).

Regarding the approach related to speckle noise, Xu et al. (2017) proposed a data augmentation technique utilizing a fusion filter-based noise suppression approach. This approach aims to address the low recognition rate and low robustness of traditional classification methods toward speckle noise. Other works have also focused on incorporating speckle noise characteristics in data augmentation techniques (Chierchia et al., 2017) and CNN models (Ma et al., 2019). Also, researchers are seeking to modify traditional CNN structures to better cater to SAR ATR requirements. These efforts include altering the learning parameters (Pei et al., 2018b), optimizing the network structure, and integrating speckle noise-related factors during model training (Kwak et al., 2019). In their work, the speckle noise was first suppressed using the fusion filter, and then the noise-suppressed images were used for network training to enhance model accuracy.

In SAR ATR tasks, CNNs have been extensively applied due to their effectiveness. Neural network structures, such as convolutional highway units, have been employed to train deeper networks with limited SAR data (Lin et al., 2017). However, it is important to consider the special characteristics of SAR images and adjust them accordingly to network models.

Although existing SAR ATR works have primarily utilized machine learning frameworks, particularly neural networks, and made significant efforts in adapting SAR images to network models, SAR images require special attention due to their uniqueness as remote sensing data. For instance, the application of deep convolutional highway units demonstrated promising results in training deeper networks with limited SAR data, the introduction of extra parameters, and the potential invalidation of layers due to shortcut connections need to be considered (Lin et al., 2017).

Literature has shown that data augmentation, particularly noise-related methods, can improve model accuracy (Ding et al., 2016). Some works have been done to simulate and incorporate speckle noise with different parameters into the training samples (Ding et al., 2016). However, evaluating handcrafted images against ground-truth data and predicting real-world recognition processes presents challenges. It is also important to consider image samples with noise cancellation in addition to noise addition, as both can contribute to the network training process.

Furthermore, to address the limitations of the CNN structure, other improvements can be considered in terms of the training process. CNNs are known for their strong feature extraction capability, resulting in success in image processing-related areas. However, when applying CNNs to SAR ATR, it is crucial to address the limited quantity of ground truth images, which are more difficult to acquire compared to optical RGB format images (Hochreiter and Schmidhuber, 1997; He et al., 2016). Overfitting can become a problem when training CNN models on SAR data.

Motivated by these considerations, this paper proposes a modified version of the Residual Network (ResNet) for SAR ATR, incorporating data augmentation to enhance recognition accuracy. Specifically, a residual strain control is introduced to modify the ResNet structure proposed by He et al. (2016), which has demonstrated superior training depth and accuracy compared to other CNNs. The proposed modification reduces training time and enlarges the SAR image dataset by both canceling and adding speckle noise, leading to improved recognition accuracy. Experimental results show that the proposed weighted ResNet, combined with data augmentation, enhances computational efficiency and recognition accuracy.

The main contributions of this paper can be summarized as:

1) This paper proposes a data augmentation method related to speckle noise in SAR images, which enhances the size and quality of the SAR image dataset. This augmentation, which involves both the addition and removal of noise, resulted in a more robust and accurate CNN model for SAR ATR.

2) A weighted ResNet is proposed which incorporates a unique residual strain control factor in its framework. By adjusting the residual strain of each weight layer, the weighted ResNet managed to enhance the model's computational efficiency, accuracy, and convergence speed, offering a major step in model optimization.

3) This paper presents comprehensive experiments to validate the effectiveness of the proposed algorithm. It further compared the weighted ResNet with other prominent CNNs, verifying its superiority in terms of training depth, model accuracy, and accelerated convergence.

The rest of the paper is organized as follows: Section 2 presents the proposed data augmentation method based on noise removal and addition. Section 3 provides details on the design of the modified residual network. Section 4 presents experimental results, while Section 5 presents the conclusions. The weighted ResNet structure includes a residual strain control factor added to the last layer of each shortcut unit. Compared with other CNNs, the improved network structure has advantages in terms of training depth and model accuracy, as well as accelerated convergence compared to the original ResNet. For data augmentation, an approach incorporating speckle noise addition and cancellation is proposed, resulting in an expanded dataset encompassing both ground-truth and noisy samples. Efficient data augmentation and improved network model accuracy in SAR ATR are achieved compared to other methods by rearranging the training and test datasets.

## 2 Data augmentation methodology

In this section, we shall present a data augmentation method based on the noise perturbation. More precisely, we augment the dataset by both canceling and adding noise.

### 2.1 Speckle noise in SAR images

It is known that SAR imaging suffers from speckle noise. Assume that the radar works under single looking mode, the observed scene can be modeled with multiplicative noise as


(1) $$\begin{array}{l} I=s\cdot n \end{array}$$

where *I* represents the observed intensity, *s* is the radar cross section (RCS) and n denotes the speckle noise. The amplitude of the RCS obeys exponential distribution with unit mean and the speckle noise is a kind of multiplicative noise. Hence, to generate a SAR image without speckle noise, we first obtain the speckle noise estimate by dividing the ground-truth images by the RCS estimate as


(2) $$\begin{array}{l} \hat{n}=I/ŝ \end{array}$$

where ŝ represents the RCS estimate obtained by applying the median filter.

### 2.2 Noise based data augmentation

**Figure 1:**
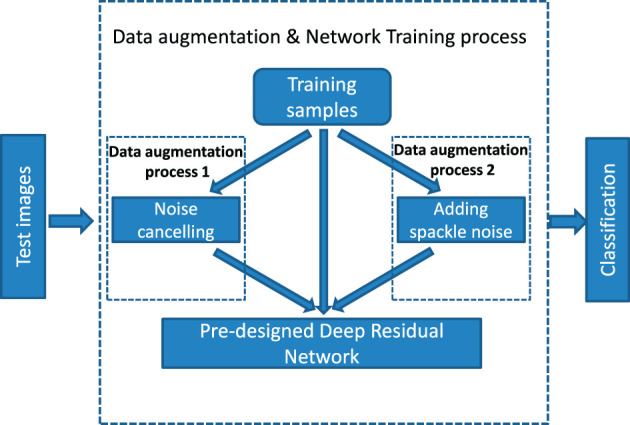
Data augmentation and network training process.

Unlike existing data augmentation approaches, we propose to expand the dataset via noise suppressing as well as noise adding. Figure 1 describes the overall system of the proposed method. It is noticed that the whole process can be in general divided into three parts: data augmentation process, model training, and classification accuracy test.

Following (1) and (2), it is not difficult to imagine that we can utilize the estimated speckle noise $\hat{n}$ to enlarge the training dataset by adding the speckle noise through multiplication and canceling suppressing through division. By doing so, it is able to get lower signal to noise (SNR) images and higher SNR images, which can be expressed as


(3) $$\begin{array}{l} I_{lowerSNR}=I\cdot \hat{n}\\ I_{higherSNR}=I/\hat{n} \end{array}$$

For data augmentation, both the lower SNR images and higher SNR images are taken as effective support.

## 3 Deep residual network design

In this section, we shall present the weighted ResNet structure, which has shortcut block units modified by introducing a residual strain control parameter in the second convolutional layer. The weighted ResNet results in less training time compared to its original counterpart.

### 3.1 Network structure unit

As evaluated in the ILSVRC 2015 classification task, ResNet achieves a 3.57% error on the ImageNet test set, which won 1st place (He et al., 2016). Equipped with shortcut connections, ResNet excels in both learning depth and recognition accuracy compared to plain convolutional neural networks. The essential idea of the ResNet is that it learns the residual function instead of the underlying mapping. The residual function, defined as the difference between the underlying function and the original intensity function (input), automatically includes reference from the input. However, in common CNN networks, the mapping function is learned as a new one in the stacked layers. In other words, the layers are reformulated as residual functions with reference to the layer inputs rather than learning unreferenced functions.

It may have overwhelming advantages, but problems also clearly exist. While conducting experiments with popular networks, we found that ResNets are less likely to converge even after other networks are well trained. This computational shortcoming drove us to explore the reason behind it and left room for improvements. Consequently, we introduced a weighted ResNet variant in our MSTAR data implementation. For a clearer explanation, the supporting theory and analysis will follow the introduction of the network structure.

**Figure 2:**
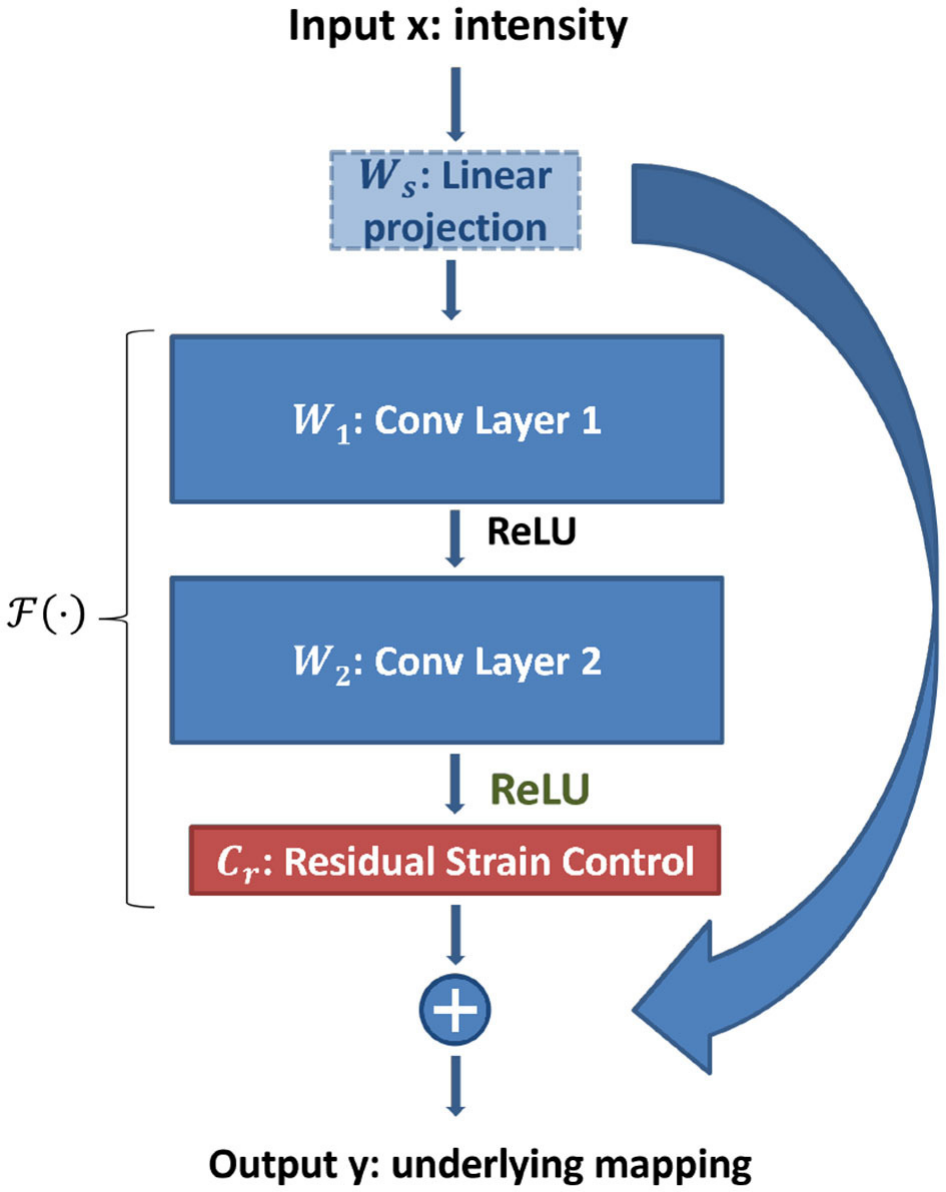
Weighted ResNet shortcut connection.

Figure 2 shows a single shortcut connection of the weighted ResNet, where the fourth and back layers are skipped for the sake of simplicity. The underlying mapping function H(x) is defined as


(4) $$\begin{array}{l} y=H\left(x\right)=F\left(x,W_{i}\right)+W_{s}x \end{array}$$

where *x* denotes the input intensity and *W*_*s*_ is a linear projection which matches the dimensions of *x* with an modified residual function *F*(·) as


(5) $$\begin{array}{l} F\left(x,W_{i}\right)=c_{\tau }\sigma \left(W_{2}\sigma \left(W_{1}x\right)\right) \end{array}$$

where σ(·) stands for the rectified linear unit (ReLU) function and the biases are omitted for simplicity, and *c*_*r*_ ∈ [−0.5, 0.5] denotes the residual strain control parameter. As can be seen from Figure 2, the residual unit is modified by adding a residual strain control after the ReLU process. During model training, the control parameter *c*_*r*_ is constrained by


(6) $$\begin{array}{l} c_{r}\leftarrow \{\begin{array}{ll} -0.5 & c_{r}+\eta \cdot \Delta c_{r}<=-0.5\\ c_{r}+\eta \cdot \Delta c_{r} & -0.5<c_{r}+\eta \cdot \Delta c_{r}<0.5\\ 0.5 & c_{r}+\eta \cdot \Delta c_{r}>=0.5 \end{array} \end{array}$$

where η is the learning rate, Δ*c*_*r*_ is the graident of parameter *c*_*r*_.

**Figure 3:**
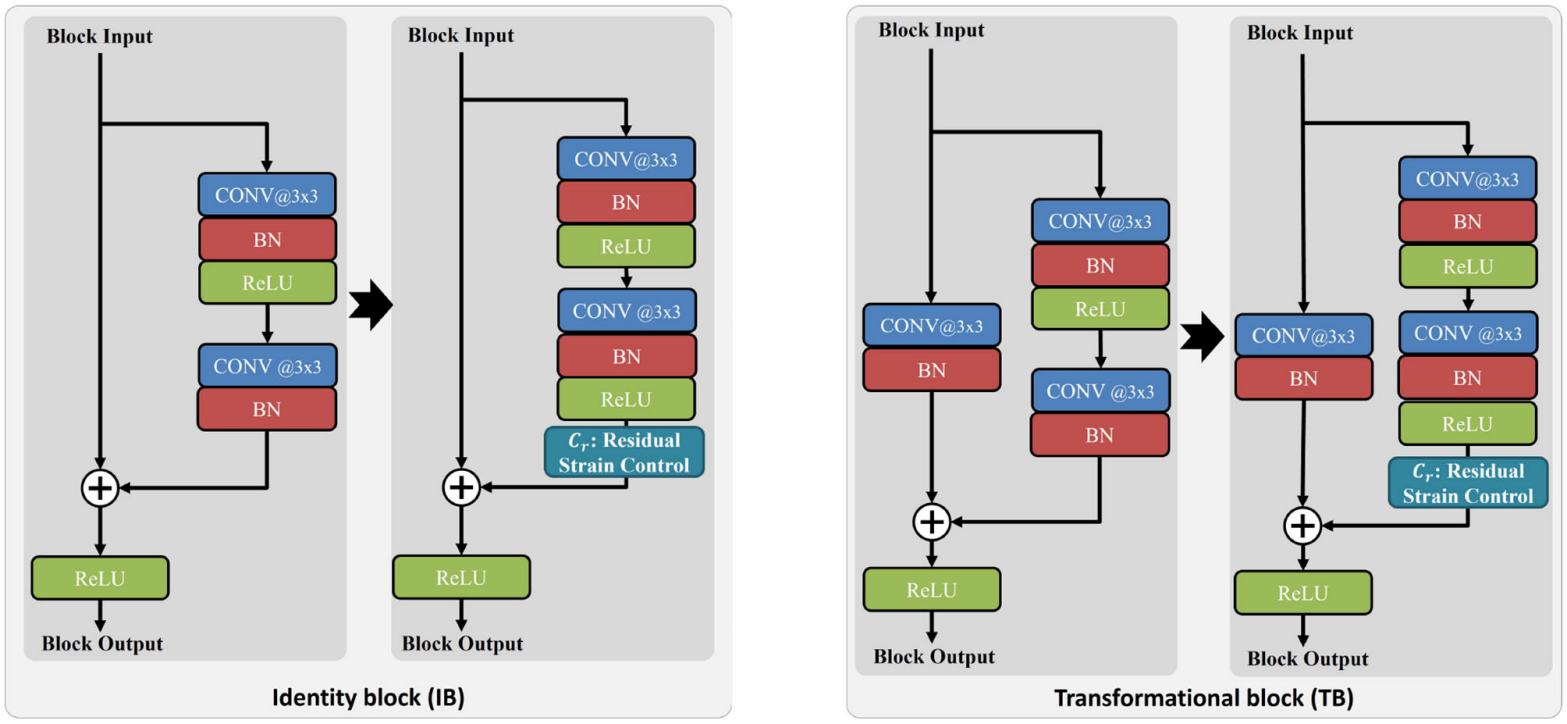
Comparison of the identity block [IB, **(left)**] and transformational block [TB, **(right)**] between basic ResNet with our proposed weighted ResNet.

Figure 3 draws a single shortcut connection of the proposed improved ResNet. Again, it can be found that, compared to the basic ResNet, the main difference is that a residual strain control unit is added. In this figure, the two blocks are termed identity block (IB) and transformational block (TB), respectively.

### 3.2 Weighted ResNet structure

In brief, the weighted ResNet involves 20 convolutional layers, in which an average pooling layer and a dense layer are the last two layers. Specifically, it takes the following form as


(7) $$\begin{array}{l} \text{Input + Conv + IB}\times \text{3 + TB + IB}\times 2\\ \text{+ TB + IB}\times \text{2 + AvgPool + Linear} \end{array}$$

**Figure 4:**
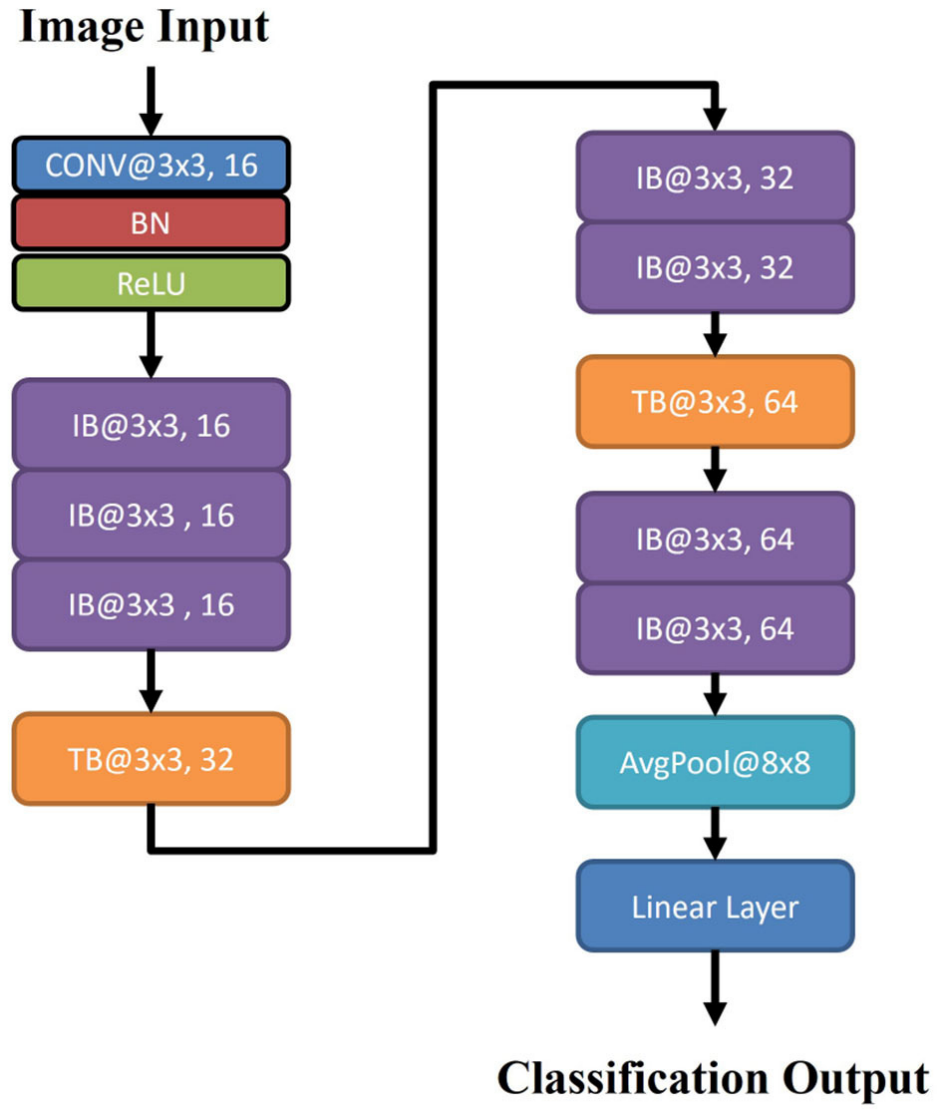
Network architecture for weighted ResNet.

The main architecture and flow chart of the weighted ResNet are given in Table 2 and Figure 4, respectively.

In weighted ResNet, a weight factor, denoted as *C*_*r*_, is introduced to the residual connections of the traditional ResNet architecture. This mechanism can assign different weights to different layers or features depending on their contribution to the final output. This allows important features to have more impact on the output and less significant features to have less impact.

The intention behind introducing a weighting mechanism varies depending on the specific application or task at hand. For example, in some contexts, introducing weights can help deal with class imbalance in the dataset. In other cases, it may be used to increase model robustness against noise or other irregularities within the data. The weights may be learned during training, using backpropagation and gradient descent, or might also be assigned based on preset criteria defined by the researchers. The methodologies can vary in different incarnations of weighted ResNet models.

### 3.3 Residual strain control for ResNet modification

Although deeper network depth and higher model accuracy are well-noticed, ResNets suffer from untoward convergence. We may first find the outstanding learning ability surprising, but it prompts further thinking and exploration post-implementation. The pain point arises when the residual information and the underlying information are merged. As observed in the Basic and Basic Inc architectures, ReLU will be applied on the residual information channel before the merger. This eventually hampers the seamless integration of the two channels. For the underlying channel, the value is in the range of (−∞, +∞), whereas the value set of the residual channel is significantly limited to merely positive after the ReLU operation. The raw merger operation in original ResNets leads to a bias far from the underlying channel, which suppresses the cognition. This will not only shorten the representation ability of networks, but also tie down the overall training process. Therefore, ResNets fall behind other CNNs in convergence inevitably.

To keep the goodness as well as speed up the training, the residual strain control parameter plays a role. As taken values in the range of [−0.5, 0.5], the residual control parameter *c*_*r*_ shifts the residual channel to both negative and positive values. And this turbocharge in turn results in a better fusion of the two channels. Significant improvements in convergence have been achieved in modified ResNets after the multiplication of *c*_*r*_.

It is worth noting that our optimization method does not add any extra structures or computational operations, thus maintaining the computational complexity, measured in FLOPS, at the same level as the base ResNet model.

### 3.4 Network training

Given the image dataset with S training samples and the corresponding ground-truth labels *x*_*i*_, *y*_*i*_, *i*∈*S*, we adopt a training cost function with $L_{2}$ regularization as


(8) $$\begin{array}{l} L=-\frac{1}{S}\underset{i\in S}{\sum }\log p_{y_{i}}\left(x_{i},\theta ,c_{r}\right)+\lambda_{1}∥\theta {∥}_{2}^{2}+\lambda_{2}∥c_{r}{∥}_{2}^{2} \end{array}$$

where *p*_*y*_*i*__ represents the predicting probability for each target class, θ is the trainable parameter of the network, λ_1_ and λ_2_ are the $L_{2}$ regularization parameters.

On the basis of the cross-entropy loss, the cost function has been equipped with two $L_{2}$ regularization factors as terms. One corresponds to the model parameters, denoted by θ, and the other to our residual strain control parameter *c*_*r*_. Here, the regularization parameters λ_1_ and λ_2_ are set to constants at training time. Although the weighted ResNet adds an upgraded structure, the training methods for minimizing its cost function and adaptively optimizing the trainable parameters are similar. We can use backpropagation for gradient computation, which has been discussed in depth in previous work. In this work, we employ one of the most popular gradient updating techniques, the momentum stochastic gradient descent (SGD) (Ruder, 2017; Tian et al., 2023) to optimize the modified residual network, which will be discussed briefly in this subsection. It is also important to note that the residual strain control parameter *c*_*r*_ is also being updated during the training process using the error back-propagation method.

SGD with momentum roots in physical law of motion to go pass through local optima. By linearly combining the gradient and the previous update, momentum maintains the update at each iteration. This keeps the update steps stable and avoids chaotic jumps. The following formulas show how SGD with momentum works:


(9) $$\begin{array}{l} \Delta \theta_{i}=\mu \Delta_{i-1}-\alpha \nabla L\left(\theta_{i}\right) \end{array}$$

(10) $$\begin{array}{l} \theta_{i}=\theta_{i-1}+\Delta \theta_{i-1} \end{array}$$

where θ_*i*_ denotes the model parameter to be estimated, Δθ_*i*_ is the ith gradient updates, μ is the momentum coefficient, α is a single learning rate, and $\nabla L\left(\theta_{i}\right)$ represents the cost function degrade. Compared with plain SGD, with the accumulating speed, the momentum SGD step will be larger than the SGD constant step. Thus, this trick will not only help to achieve global minimum but also increase robustness.

## 4 Experiments

### 4.1 Dataset

**Figure 5:**
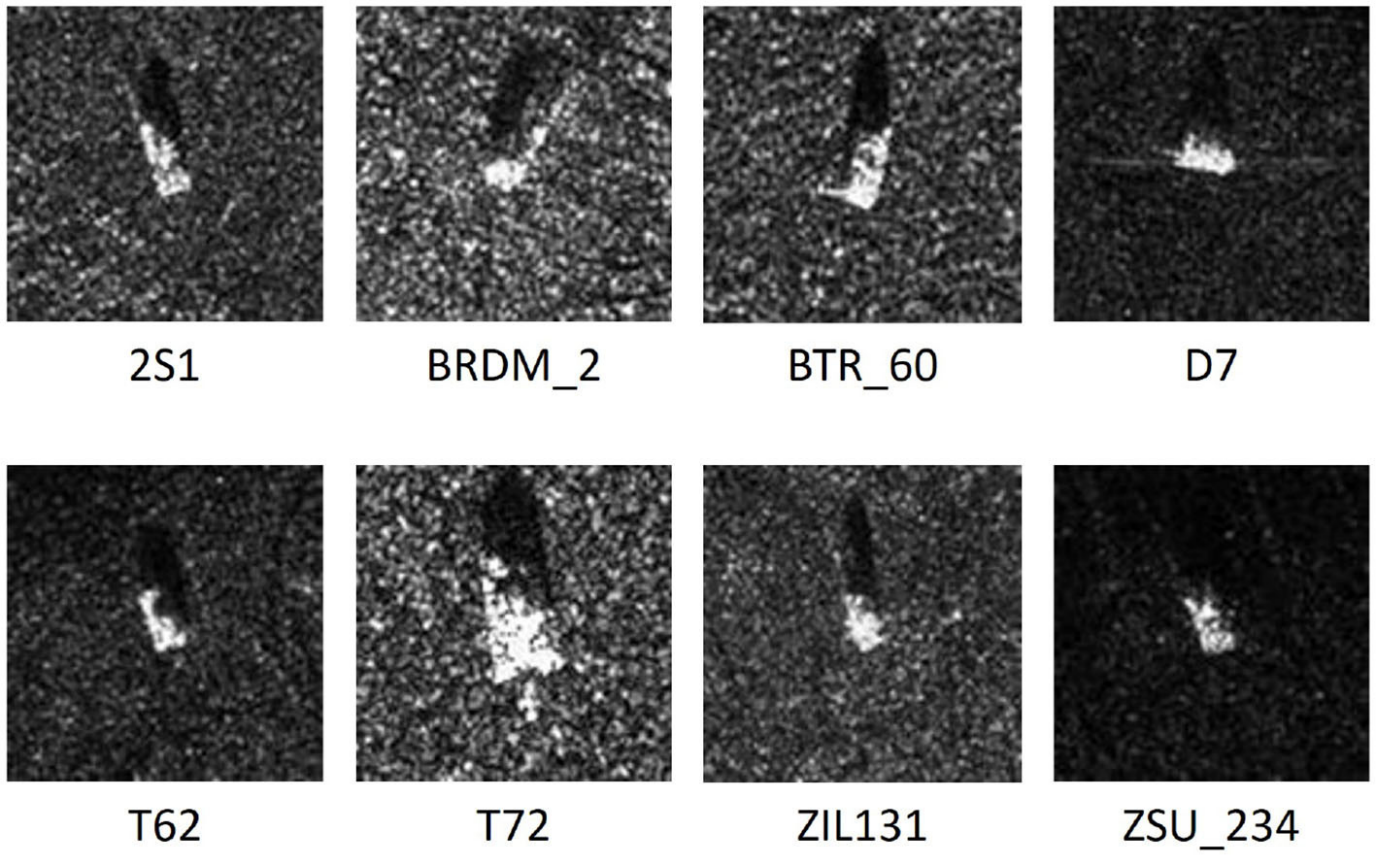
Example SAR images of the eight different classes.

We evaluate our proposed method using a benchmark dataset from the Moving and Stationary Target Acquisition and Recognition Program published (Zhao and Principe, 2001) by the US Defense Advanced Research Projects Agency and the US Air Force Research Laboratory. The dataset consists of X-band SAR images of different types of military vehicles (e.g., APC BTR60, Main Tank T72, and Bulldozer D7) with elevation angles of 15° and 17°. The image resolution is 0.3m × 0.3 m, some example images of different classes are shown in Figure 5.

To train the weighted ResNet, all the images we used in our experiments are cropped to 100 × 100 pixels, with the target located at the center. We primarily use eight types of target images, and the number of images used for training and testing is listed in Table 1. The cropped image dataset contains 8 types of military ground targets, namely T62, BRDM2, BTR-60, 2S1, D7, ZIL131, ZSU-234, and T72. Images of each target are collected at depression angles of 15° and 17° and then turned at an angle of 360°. We note that one uses images with a depression angle of 15° for training and images with 17° for testing. However, this may shorten the recognition ability of the trained deep learning network because of the missing spatial information that could have been included. We stick with this idea and do training experiments with images of 15° and 17° with depression angle.

**Table 1:** List of noise perturbed SAR images (data augmentation).

**Target**	**Noise canceled 3 × 3**	**Noise canceled 5 × 5**	**Noise canceled 7 × 7**	**Noise added *M* = 0.5**	**Noise added *M* = 1.0**	**Noise added *M* = 1.5**	**Total**
2S1	573	573	573	573	573	573	3, 438
T62	572	572	572	572	572	572	3, 432
BRDM2	572	572	572	572	572	572	3, 432
BTR-60	451	451	451	451	451	451	2, 706
D7	573	573	573	573	573	573	3, 438
ZIL131	573	573	573	573	573	573	3, 438
ZSU-234	573	573	573	573	573	573	3, 438
T72	573	573	573	573	573	573	3, 438
Total	3,887	3,887	3,887	3,887	3,887	3,887	23,322

In order to expand the capacity of the original dataset by removing and adding noise (different filtering or noise distribution parameters), in our experiments, we use cropped images of 8 targets to generate image variants, and 400 images are randomly selected for each target.

**Figure 6:**
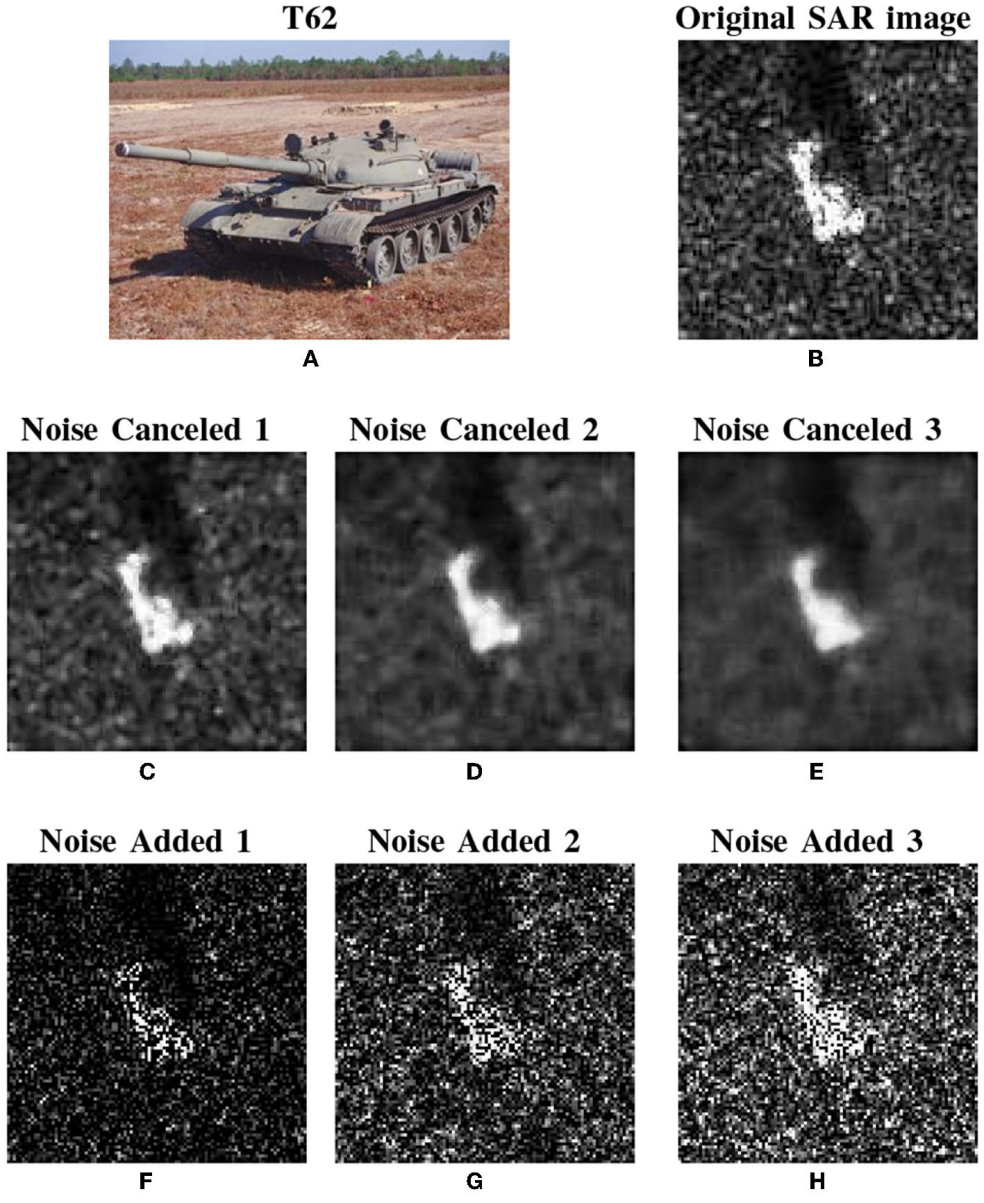
The original optical image **(A)** and SAR image **(B)**, and noise perturbed SAR images **(C–H)**.

For illustration purposes, we take one of the T62 SAR images as an example to demonstrate the noise removing and adding behaviors. Figures 6A, B show the original optical image and the SAR image. Figures 6C–E draw the noise-removing images generated through median filtering with the templates of 3 × 3, 5 × 5, and 7 × 7, respectively. Figures 6F–H depict the noise-added images with multiplied exponentially distributed speckle noise with means (termed as *M*) of 0.5, 1.0, and 1.5, respectively. Finally, the whole noise canceled and added images generated from the cropped images are listed in Table 1. According to our design, the SSIMs for the filters of both noise removal and noise adding are set by 90%, 82.5%, and 75%, respectively.

### 4.2 Classification results

We first conducted experiments to validate our proposed speckle noise-based method. The confusion matrix of our weighted ResNet can be found in Tables 2, 3 as comparisons of data augmentation. The classification accuracy of weighted ResNet using non-augmented training data is 94.56% (7,269/7,680). Table 2 shows the confusion matrix of weighted ResNet using non-augmented training data. Each row in the confusion matrix represents the actual target class, and each column denotes the class predicted by the weighted ResNet. The classification accuracy of weighted ResNet using augmented training data is 99.65% (7,653/7,680). Table 3 shows the confusion matrix of weighted ResNet using augmented training data. Each row in the confusion matrix represents the actual target class, and each column denotes the class predicted by the weighted ResNet.

**Table 2:** Confusion matrix of the weighted ResNet (without data augmentation).

	**T62**	**BRDM-2**	**BTR-60**	**2S1**	**D7**	**ZIL131**	**ZSU-234**	**T72**	**Total**
T62	895	10	0	3	24	15	13	0	93.23
BRDM-2	13	930	0	6	1	10	0	0	96.88
BTR-60	6	12	891	4	36	0	9	2	92.81
2S1	6	3	8	909	10	21	0	3	94.69
D7	0	0	20	0	938	0	2	0	97.71
ZIL131	19	3	28	5	0	890	1	14	92.71
ZSU-234	3	16	15	0	9	0	917	0	95.52
T72	0	0	32	0	16	10	3	899	93.64
Total	–	–	–	–	–	–	–	-	94.56

**Table 3:** Confusion matrix of the weighted ResNet (with data augmentation).

	**T62**	**BRDM-2**	**BTR-60**	**2S1**	**D7**	**ZIL131**	**ZSU-234**	**T72**	**Total**
T62	954	2	0	1	0	0	3	0	99.38
BRDM-2	0	960	0	0	0	0	0	0	100.00
BTR-60	3	0	953	2	0	1	0	1	99.27
2S1	0	0	0	958	0	2	0	0	99.79
D7	0	0	0	0	960	0	0	0	100.00
ZIL131	3	4	0	3	0	950	0	0	98.96
ZSU-234	0	0	2	0	0	0	958	0	99.79
T72	0	0	0	0	0	0	0	960	100.00
Total	–	–	–	–	–	–	–	–	99.65

**Figure 7:**
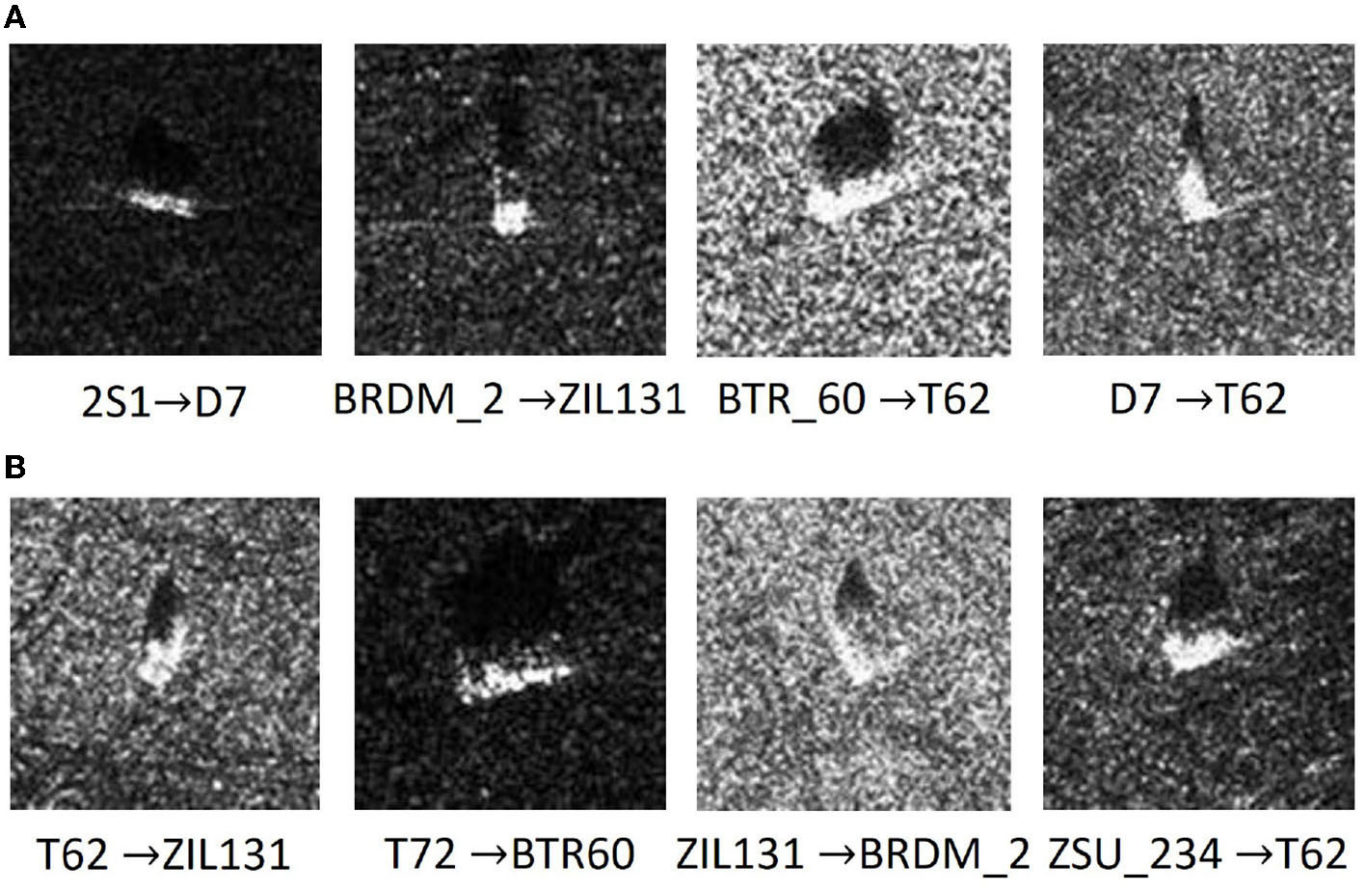
Examples of misclassified samples in each category, with only one example selected per category. The text below the image, *A*→*B*, signifies that the expected category is **(A)**, but the model mistakenly classified it as **(B)**.

The classification accuracy of weighted ResNet with data augmentation is up to 99.65%, increasing by almost 5.1%. Additionally, the weighted ResNet structure has a relatively lower classification performance on the ZIL131 (92.71%) and BTR-60 (92.81%), followed by T62 (93.23%). After the dataset extension, the classification accuracy of ZIL131 is up to 98.96%. A similar improvement is seen in the BTR-60 and T62, each with nearly a 5% increment. This indicates that the speckle noise perturbation based data augmentation method is valid. Moreover, the recognition rate of armored personnel carriers is relatively low, which suggests that the distribution of those targets is near in the feature space. The above results are consistent with the trends observed in which has been published in Kang et al. (2017), a contributor in SAR ATR feature exaction. Further, in Figure 7, we show some instances of misclassification, where we selected only one example from each category for presentation. *A*→*B* means cases where a sample with the label *A* is incorrectly classified as *B* by the model.

### 4.3 Network performance comparsion

In our experiments on weighted ResNet and ResNet, the following setups are applied: the mini-batch size is 128, the epoch number is 160, the dynamic learning rates are 1.0 for the first 80 epochs, 0.1 for the next 40 epochs and 0.01 for the remaining epochs, the momentum coefficient starts from 0.9. For weighted ResNet and ResNet, the L2 regularization parameters are 0.0001. In addition, taking into account the model difference between AlexNet and VGG networks, the training parameters designed for AlexNet and VGG are: mini-batch size 128, epoch number 200, initial momentum coefficient 0.9, and regularization parameters 0.0005. The dynamic learning rates for AlexNet are 0.1 for the first 25 epochs and 0.0001 for the remaining epochs, while the learning rates for VGG are 0.1 for the first 20 epochs and 0.01 for the next 20, then 0.001 for the next 20 epochs and 0.0001 for the following rest. One may notice that we picked the learning rate by 1.0 for the first 80 epochs in training weighted ResNet, which is much higher than what had been shown in previous literature. The reason is that we took advantage of momentum SGD in network training. Momentum SGD is not sensitive to learning rate mis-specification or curvature variance, and will tolerate a relatively wide range of learning rates. Thus no unusual signs were observed during the training process. Another reason may refer to the experiences gained while conducting network training experiments on different network structures with large volumes of other data sets. Here we train the ResNet and weighted ResNet without loading pre-trained models. The method is robust against noise and momentum SGD training will skip local optimal solutions.

In order to illustrate its advantages, the weighted ResNet is compared to its original counterpart (He et al., 2016), SVM (Zhao and Principe, 2001), A-convNet (Chen et al., 2016), and Ensemble CNN (Lin et al., 2017), CNNs [(Morgan, 2015; Ding et al., 2016; Furukawa, 2017), as well as other two deep neural networks [AlexNet (Krizhevsky et al., 2012) and VGG16 (Simonyan and Zisserman, 2014)] for SAR image classification. As shown in Table 4, there is a 0.81% accuracy rise for CNN-3, while nearly 3.57% on AlexNet, and over 4% increase noted in VGG16, ResNet, and weighted ResNet. Table 4 clearly shows that ResNet has a higher recognition accuracy than other networks. Other modified networks without data augmentation can achieve accuracy over 99% (Chen et al., 2016; Lin et al., 2017).

**Table 4:** Accuracy comparison with other methods.

**Method**	**Without data augmentation**	**With data augmentation**
SVM (Zhao and Principe, 2001)	90.00%	–
Ensemble CNN (Lin et al., 2017)	99.09%	–
A-convNet (Chen et al., 2016)	99.13%	–
CNN-1 (Ding et al., 2016)	–	94.56%
CNN-2 (Morgan, 2015)	–	92.30%
CNN-3 (Furukawa, 2017)	98.75%	99.56%
AlexNet (Krizhevsky et al., 2012)	93.71%	97.28%
VGG16 (Simonyan and Zisserman, 2014)	94.12%	98.97%
ResNet (He et al., 2016)	94.58%	99.65%
Weighted ResNet	94.65%	99.65%

**Figure 8:**
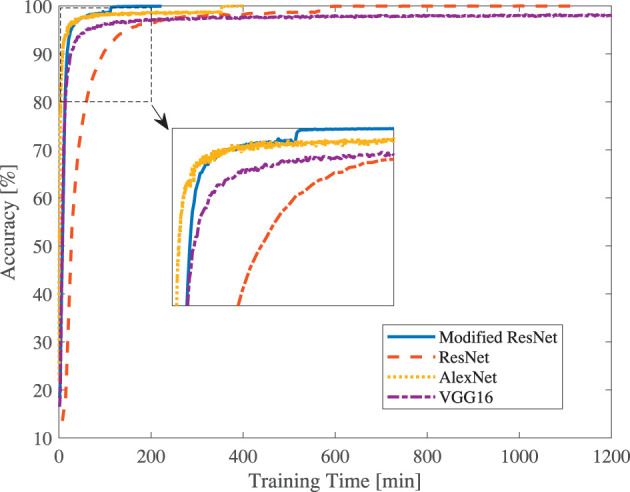
Comparison of the accuracies vs. training time.

Recognition accuracies along with training time curves are displayed in Figure 8. Without a shortcut connection structure, the AlexNet and VGG can converge much faster than ResNet. The original ResNet (400 min) takes nearly twice the time of AlexNet and nearly four times the VGG. While weighted ResNet (165 min) easily passes over VGG and nearly catches AlexNet, which is delightful. It should be pointed out that although the weighted ResNet does not provide an ultimate accuracy improvement, it can considerably shorten the training time as demonstrated in Figure 8. In fact, in the case of limited training time, e.g., <400 min, the weighted ResNet achieves the highest recognition accuracy among the networks we have tested.

## 5 Discussion and conclusion

In this paper, we presented a weighted ResNet model for SAR ATR. Our method tackled problems usually associated with conventional CNN models such as overfitting due to the constrained quantity of ground truth images and the unique complexities presented by speckle noise in SAR images. We incorporated data augmentation and introduced a distinctive residual strain control method, which together contributed to the generation of a weighted ResNet with increased computational efficiency, boosted recognition accuracy, and faster convergence. The data augmentation method proposed in this paper, which involved the addition and cancellation of speckle noise, successfully expanded the quality and size of the SAR image dataset and made the model more resilient. This step was critical, as it provided a practical solution to the issue of scarce ground truth images.

Our novel introduction of a residual strain control to adapt the ResNet model contributed to significant improvements in model efficiency and recognition accuracy and reduced training time. It efficiently managed the residual strain of each weight layer, leading to faster convergence and improved optimization.

Experimental results displayed the superiority of our proposed weighted ResNet model when compared to other prominent CNNs. The accelerated convergence, remarkable training depth, and improved model accuracy showcased our model's effectiveness and robust capabilities in SAR ATR.

While our research and results are promising, the continuous advancement in AI and deep learning applications will consistently present avenues for growth. Future work can focus on further enhancements of the weighted ResNet model for improved model stability and generalization capabilities. Additionally, exploring more sophisticated data augmentation techniques can help in producing even more robust models capable of handling different SAR ATR scenarios. Applying the developed model to other similar imaging techniques can also be an interesting aspect to look into.

## Data availability statement

The original contributions presented in the study are included in the article/supplementary material, further inquiries can be directed to the corresponding author.

## Author contributions

JL: Conceptualization, Data curation, Formal analysis, Writing – original draft. CP: Conceptualization, Funding acquisition, Methodology, Supervision, Writing – review & editing.
